# On the Formation of Nanogratings in Commercial Oxide Glasses by Femtosecond Laser Direct Writing

**DOI:** 10.3390/nano12172986

**Published:** 2022-08-29

**Authors:** Qiong Xie, Maxime Cavillon, Diego Pugliese, Davide Janner, Bertrand Poumellec, Matthieu Lancry

**Affiliations:** 1 Institut de Chimie Moléculaire et des Matériaux d’Orsay (ICMMO), Université Paris-Saclay, CNRS, 91405 Orsay, France; 2 Department of Electronics and Telecommunications, Politecnico di Torino, 10129 Torino, Italy; 3 Department of Applied Science and Technology (DISAT) and RU INSTM, Politecnico di Torino, 10129 Torino, Italy

**Keywords:** nanogratings, birefringence, femtosecond laser direct writing, alkali, viscosity

## Abstract

Nanogratings (NGs) are self-assembled subwavelength and birefringent nanostructures created by femtosecond laser direct writing (FLDW) in glass, which are of high interest for photonics, sensing, five-dimensional (5D) optical data storage, or microfluidics applications. In this work, NG formation windows were investigated in nine commercial glasses and as a function of glass viscosity and chemical composition. The NG windows were studied in an energy—frequency laser parameter landscape and characterized by polarizing optical microscopy and scanning electron microscopy (SEM). Pure silica glass (Suprasil) exhibits the largest NG window, whereas alkali borosilicate glasses (7059 and BK7) present the smallest one. Moreover, the NG formation windows progressively reduced in the following order: ULE, GeO_2_, B33, AF32, and Eagle XG. The NG formation window in glasses was found to decrease with the increase of alkali and alkaline earth content and was correlated to the temperature dependence of the viscosity in these glasses. This work provides guidelines to the formation of NGs in commercial oxide glasses by FLDW.

## 1. Introduction

Type II modifications, generally characterized by the formation of self-assembled periodical nanostructures known as nanogratings (NGs), were first shown and studied in silica glass [1]. Such NGs, photo-induced in bulk glasses irradiated by femtosecond (fs) laser, have attracted high interest in the past few years due to their peculiar properties such as linear/circular birefringence and dichroism, extraordinary thermal stability, selective chemical etching, etc. These features have enabled applications such as 5D optical data storage, 2D and 3D space-variant birefringent devices, sensors in the harsh environment, microfluidic channels, etc. [2,3,4,5,6,7]. Femtosecond laser direct writing (FLDW) can create permanent modifications strongly localized in 3D while focusing inside transparent materials, arising from nonlinear absorption phenomena at the root of the laser-matter interaction process.

These nanostructures are made of porous nanolayers mostly observed in silica and silica-rich glasses [8,9]. In our early work, the formation of these nanopores was associated with a tensile stress-assisted oxide decomposition [10] that was recently revisited as a nanocavitation mechanism [11,12]. However, until now, the influence of the glass physical properties (melting temperature, thermal diffusivity, absorption) on the NG formation process has been significantly investigated, but without rationalization yet. Such analysis would enable the tailoring of the chemical composition and structure of glasses including nanoporous silica to define the best laser conditions for a targeted application (e.g., reducing the required number of pulses for optical data storage or increasing the writing speed for writing birefringent optics). As part of the task, different dopants of silica glass have been analyzed in the context of NG formation, namely, germanium, phosphorus, fluorine, or chlorine [10,13,14,15]. During the last two decades, nanostructures have been found inside a handful of materials: fused silica, GeO_2_ [16,17,18], sapphire [19], tellurium oxide [20], quartz [21], ULE glass [9,22], and even in some types of alumino-borosilicate glasses [9,22,23]. The influence of alkali cations in a silicate matrix was also proven to be detrimental on the formation of NGs [24,25]. Among this list, fused silica is the most common material to induce NGs. These were also found in porous silica prepared from phase-separated alkali-borosilicate glass by removing the borate phase in a hot acid solution [3]. Even twin or single isolated nanoplanes can be induced by controlling laser pulse energy [26], which could be used to write nanofluidic channels [3,27,28]. Silica provides a relatively large processing window in contrast to other materials. However, the highest retardance was obtained in porous silica glass with a porosity size in the range of 2.5 to 5 nm, also providing Type II modifications with a limited amount of stress [29,30]. The NG formation is also expected to be enhanced in glasses with a high free volume or with compositional fluctuations at the nanoscale, which could act as a precursor for the nanostructure formation.

This work provides insights into the relative difficulty of forming NGs in a wide range of common commercial oxide glasses. The main objective is to identify the so-called Type II windows (i.e., NGs windows) in them, and thus the ability for a glass to imprint NGs but also to provide some indicative performances in terms of energy consumption, writing speed, and birefringence amplitude. The selection of optical glasses investigated covered Suprasil, ULE, B33, AF32, Eagle XG, 7059, BK7, soda-lime, and GeO_2_, and for some of them, preliminary results have been published in [12]. They all present interests in diverse applications such as flat glass for display and silicon wafer assembly (Eagle XG, AF32), glass substrate for electronic components (7059), infrared photonic devices (GeO_2_), precision optics for medical technologies, photovoltaic or space telescope substrates (Borofloat33), visible and near-infrared prisms and micro-optic elements (BK7). The chosen glasses were then fs-irradiated using similar experimental conditions. This systematic study provides a direct comparison between a wide range of multicomponent glasses and their respective NG existence windows in the energy—repetition rate landscape. In addition to polarized microscopy, complementary scanning electron microscope (SEM) analysis enables the existence of NGs to be proven through the observation of nanopores organized into quasi-periodic planes. Moreover, by determining the laser track length containing the nanopores, the quantitative birefringence value of NGs can be determined. Then, the results in terms of the alkali or alkaline earth cation content on the formation of NGs and related performances were rationalized. Finally, our recent viscosity-based approach [12] was exploited and interestingly revealed that the energy—repetition rate landscape is well-correlated to the predicted NG existence domain.

## 2. Materials and Methods

All of the glass samples employed in this work were directly acquired from glass makers, except for GeO_2_, which was prepared in the laboratory. For this purpose, a powder mixture of 30 g per batch composed of GeO_2_ (99.9%, Serlabo technologies, Entraigues-sur-la-Sorgue, France) was placed inside a platinum crucible, dried at 200 °C for 2 h, and finally melted at 1400 °C for 1 h using a heating rate of 10 °C/min. Finally, the molten mixture was quenched between two metal plates preheated at around 350 °C. Glass samples were selected, cut, and polished to an optical grade quality. All of the glass compositions are reported in Table 1.

A commercial Yb-doped fiber amplifier femtosecond laser (Satsuma, Amplitude Systèmes Ltd., Pessac, France) was used to irradiate each sample, with a laser-operating wavelength centered at 1030 nm and a fixed pulse duration of 800 fs. The laser beam was focused using a 0.6 numerical aperture (NA) aspheric lens (estimated beam waist *w*_0_ ~1.5 µm) at a depth of 300 µm (in air). The sample was placed on an XYZ-motorized translation stage, and the direction of the linear polarization was controlled using a *λ*/2 waveplate mounted on a rotation stage. The inscription patterns inscribed in the samples were a series of rectangles, which were composed of a series of parallel lines or single lines. For most glasses, a constant pulse density *f*/*v* = 1000 pulses/μm was used, with *ƒ* being the pulse repetition rate (Hz, or pulses/s) and *v* is the scanning speed (μm/s). *ƒ* was varied between 10 and 1000 kHz; therefore, the laser *v* was varied from 10 to 1000 μm/s accordingly. The pulse energy *E* was varied from 25 nJ to 4 μJ, thus the different modification thresholds were identified [13]. Two different laser polarization orientations were used to write lines: perpendicular (called Xy) and parallel (called Xx) to the laser scanning direction (*X*-axis). This was then used (1) to highlight the formation of polarization dependent birefringence, which is a key characteristic feature of NG formation [31] and (2) to investigate the nanostructures using SEM.

Optical retardance (*R*) of the laser-induced modifications, defined as the product of linear birefringence (*LB*) by the thickness (*l*) of the birefringent object (i.e., *R* = *LB* × *l*) was measured using an Olympus BX51 polarizing optical microscope equipped with a “de Sénarmont” compensator. This compensator coupled a high-precision quarter waveplate with a 180° rotating analyzer to provide retardation measurements in the visible range. Such a setup had an accuracy that approached a few nm when used in our conditions.

In order to investigate the morphology and texturing of Type II modifications, the two different polarization laser modification lines were cleaved perpendicularly to their writing direction, which allowed the laser track cross-sections to be observed by SEM (Field-Emission Gun Scanning Electron Microscope, ZEISS SUPRA 55 VP, 1 kV accelerating voltage). Depending on the laser polarization orientation (i.e., Xx or Xy configurations as described above), the inside of the nanoplanes (Xx) or the periodicity of the nanoplanes (Xy) could be observed.

## 3. Results and Discussion

### 3.1. Study of Type II Modifications Windows in the Different Glasses

**Figure 1:**
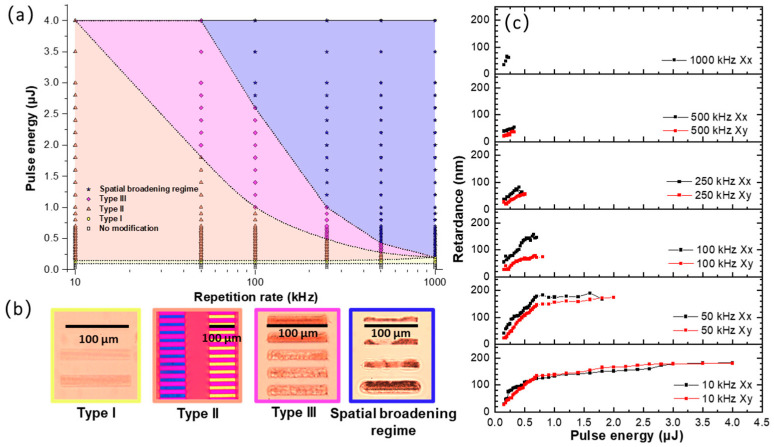
(**a**) Processing windows for the different types of fs-laser modifications in Borofloat33 (B33) glass. (**b**) Optical microscope images of the different types of modifications. (**c**) Retardance measured within the Type II regime by the Sénarmont technique. Experimental conditions: 1030 nm, 800 fs, 0.6 NA, from 0.025 to 4 µJ, pulse density *f*/*v* = 10^3^ pulse/µm, Xx and Xy writing configurations.

Different types of modifications induced by fs-laser in glass, reported in the literature as IR-fs Type I, Type II and Type III, were observed in the irradiation landscape (energy—repetition rate). Figure 1a,b shows these modifications in Borofloat33 (B33) glass as an example, along with the corresponding laser parameters and optical microscopy characterization. At very low energies (≤0.10 μJ), no permanent modifications were detected in the glass. As the energy progressively increased (moving vertically up in Figure 1a), the appearance of the Type I modification was identified. Type I corresponds to a permanent isotropic refractive index change (either positive or negative depending on composition) and is typically found at low fluence values. The corresponding optical microscope image is shown in Figure 1b using a transmitted light observation configuration. As the energy is increased, the Type II regime is established. The corresponding optical microscope image, taken under a first-order full waveplate placed at 45° of a crossed analyzer/polarizer, allowed us to reveal the formation of polarization dependent linear birefringence (i.e., the slow axis orientation rotates almost linearly with the laser polarization), a characteristic feature of NGs. It is worth pointing out that the Type II window becomes smaller at higher repetition rates (*f*).

By further looking at Figure 1a, the Type III regime appeared at higher energies, the laser tracks looked inhomogeneous, and voids issued from the micro-explosions were erratically induced inside the irradiated affected volume. As the repetition rate increased, the Type III threshold and its processing window decreased. Finally, heat effects occurred at a high repetition rate and energy, whose optical signature was mostly related to the strong spatial extension of the laser tracks (beyond the light beam size itself) accompanied by black areas, as reported in Figure 1b. This spatial broadening is related to the thermal effects (i.e., thermal diffusion coefficient and related thermal diffusion time *τ_th_*), but one could find two different origins [32].

In the following, it was assumed that a temperature effect was at the origin of the spatial broadening. At low repetition rates (typ. 1/*f >> 7τ_th_* with *τ_th_* ≈ *ω*_0_^2^/(4*D_th_*), where *w*_0_ is the diameter of the energy source at 1/e), the temperature elevation increases with the deposited energy. Thus if one considers that line broadening is related to the overcoming of a local temperature (let us say a “transformation temperature”), the increase in the temperature with the deposited energy indeed progressively enlarges the spatial volume of the transformation, corresponding here to a permanent refractive index change. On the other hand, at high repetition rates and high energy, a well-known heat accumulation progressively occurs [33], meeting the condition where the time between two consecutive pulses is of the order of the thermal diffusion time, typ. 1/*f < 7τ_th_* (corresponding here to a 10% increase in the average temperature [32]). In these conditions, the imprinted width for a given temperature can overcome the light energy source size ($w_{r}$ and $w_{z}$) and usually scales up with the increasing repetition rate.

Figure 1c shows the optical retardance (*R*) writing energy dependence, measured within the Type II regime by the Sénarmont compensator technique for two writing polarization configurations. In B33, a larger *R* was found at 10 and 50 kHz. Usually, *R* increases monotonously with the energy before 0.75 µJ and then exhibits a trend to saturation at higher energies. The maximum *R* values at “saturation” decreased with an RR increase except for 10 kHz. Finally, at higher energies, another regime was established and the retardance dropped down. Note that the retardance of Xx was a little bit larger than for the Xy writing configuration, a feature commonly observed in the literature.

**Figure 2:**
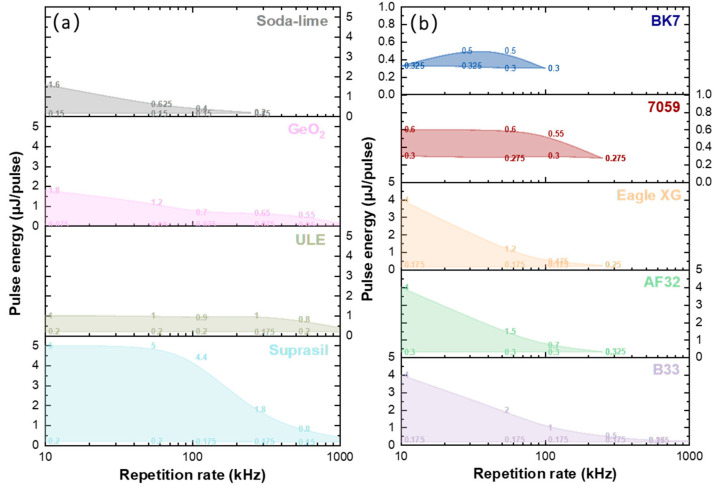
(**a**) Type II modification windows in the Suprasil, ULE, GeO_2_, and soda-lime glasses. (**b**) Type II modifications windows in B33, AF32, Eagle XG, 7059, and BK7 alumino-borosilicate glass. (Zoom × 5 in the figure of 7059 and BK7 samples). Experimental conditions: 1030 nm, 800 fs, 0.6 NA, pulses densities *f*/*v* = 10^3^ pulse/µm (Suprasil, GeO_2_, AF32, B33, Eagle XG), *f*/*v* = 10^4^ pulse/µm (soda-lime), *f*/*v* = 10^5^ pulse/µm (7059 and BK7), Xx writing configuration.

Figure 2 shows a comparison of the Type II modification windows for different commercial oxide glasses for quite similar writing conditions. Here, the ratio between the writing speed and repetition rate was kept fixed at *f*/*v* = 10^3^ pulse/µm for most glasses, especially with the high silica content and GeO_2_ glass. However, more pulses were needed to imprint NGs in the alkali silicate glasses, namely 10^4^ pulse/µm for soda-lime glass and 10^5^ pulse/µm for 7059 and BK7. This is the first report of the effective imprinting of NGs in alkali-rich glasses such as BK7, Corning 7059, or soda-lime glasses.

From Figure 2a, it can be noticed that the pure silica glass (Suprasil) had the largest window related to the existence of NGs, whereas it was the opposite for the borosilicate (BK7) glass; this is likely to be due to its high alkali content but also its high B_2_O_3_ content. Additionally, only the high silica content glasses (Suprasil, ULE, B33) and GeO_2_ glasses showed the NG formation above 250 kHz. Then, by comparing the alkali free or low alkali content alumino-borosilicate (AF32, B33 and Eagle XG) glasses with the alkali borosilicate (7059 and BK7) or soda-lime glasses, larger Type II windows were found for the former. In contrast, it was very difficult to imprint NGs in the 7059 and BK7 glasses; a large number of pulses, typ. > 10^5^ pulse/µm, needs to be cumulated, in agreement with previous reports in the literature concerning Na_2_O-SiO_2_ [25] or Na_2_O-GeO_2_ [10] glasses.

### 3.2. Study of Type II Modifications Origin in the Different Glasses

**Figure 3:**
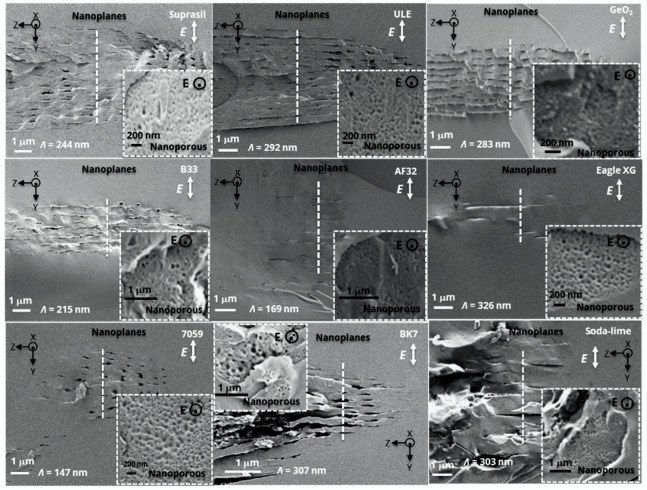
The SEM secondary electron micrographs of laser track cross-sections written in different oxide glasses (Suprasil, ULE, GeO_2_, B33, AF32, Eagle XG, 7059, BK7, and soda-lime). The polarization direction (*E*) was perpendicular or parallel (insert) to the writing direction. Experimental conditions: 1030 nm, 800 fs, 0.6 NA, pulses densities *f*/*v* = 10^3^ pulse/µm (ULE, GeO_2_, AF32, B33), *f*/*v* = 10^4^ pulse/µm (soda-lime, Eagle XG), *f*/*v* = 10^5^ pulse/µm (50 kHz for 7059 and 25 kHz for BK7), 250 fs, *f*/*v* = 500 pulse/µm (Suprasil), Xx writing configuration. The pulse energy was chosen to fall within the Type II regime.

In this section, the presence of Type II nanostructures was investigated by SEM imaging. A micrograph with both parallel and perpendicular writing polarization conditions is provided for each sample in Figure 3, showing the NG structures present in each investigated commercial glass. Our observation technique, based on cleaving the sample, is likely to not be the best way to preserve NGs, but it allows one to easily see the nanopores (in the Xx configuration). First, the nanolayers were found to be quasi-periodic and oriented along the laser polarization direction in agreement with the overall literature. Second, all of them appeared to be nanoporous, thus revealing that a glass oxide decomposition process occurred in all of these compositions, highlighting in such a way that this is a general mechanism. It is worth noting that the nanolayers appeared quite “disrupted” in our conditions. However, based on the literature, a high pulse number or large pulse density [34] will increase the uniformity of the NGs. This needs a low writing speed rather than a high repetition rate to avoid a too high temperature. It is also known that high pulse energy will lead to disrupted NG formation [9], whereas long pulse duration (typ. around 800 fs) [35] makes the NGs more uniform. Key applications where homogenous NGs are needed are micro-optics and optics such as 3D geometric phase optics and 3D space variant birefringent devices, whereas sensing applications and optical data storage do not necessarily require homogeneous NGs. In addition, recent work has indicated that using a Type X regime (individual elongated nanopores) [36] in a multilayer strategy looks to be a promising approach to develop optics and optical storage with much lower optical losses.

**Figure 4:**
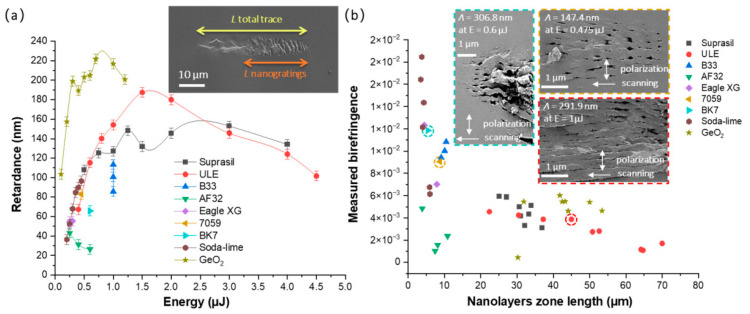
(**a**) The measured optical retardance *R* and (**b**) corresponding birefringence (*B* = *R*/*L_nanogratings_*) of different glasses (insets are the SEM images of BK7, 7059, and ULE). Experimental conditions: 1030 nm, 800 fs, 0.6 NA, pulses densities *f*/*v* = 10^3^ pulse/µm (ULE, GeO_2_, AF32, B33), *f*/*v* = 10^4^ pulse/µm (soda-lime, Eagle XG), *f*/*v* = 10^5^ pulse/µm (50 kHz for 7059 and 25 kHz for BK7), 250 fs, *f*/*v* = 500 pulse/µm (Suprasil), Xx writing configuration.

From the SEM micrographs, the thickness *L_total trace_* of each laser track was measured for both writing configurations. More specifically, the length over which porous NGs were observed along the laser propagation direction, *L_nanogratings_*, was determined. From these values, the birefringence *LB* was calculated using the retardance values using the following expression *R* = *LB* × *L_nanogratings_*. This is exemplified in the inset of Figure 4a. The modified NG structure is clearly visible, and the period (*Λ*) is much smaller than the laser wavelength (see the insets in Figure 3 and Figure 4b).

In this view, the retardance writing kinetics of all these samples were thus measured according to the pulse energy and in similar writing conditions (1030 nm, 800 fs, 0.6 NA, 50 kHz). From Figure 4, it can be assessed that the glasses with a strong 3D network (e.g., tectosilicates), namely, ULE, Suprasil, and GeO_2_, displayed a larger retardance. However, the corresponding birefringence was smaller since they had a quite long nanolayer zone, typ. up to 70 μm at high energy. In contrast, the alumino-borosilicate glasses (B33, AF32, Eagle XG, BK7, and 7059) showed smaller retardance at low energy and a shorter nanolayer zone length, but the related birefringence was relatively larger. This indicates that the NGs had a strong refractive index in contrast to these glasses, which might reveal not only the nanopore formation, but also the nanoscale phase separation. Among all of the commercial glasses, soda-lime possessed similar retardance values at low energy compared to silica (Suprasil), but exhibited the largest birefringence.

### 3.3. Influence of Alkali Content on NG Windows in Different Glasses

**Figure 5:**
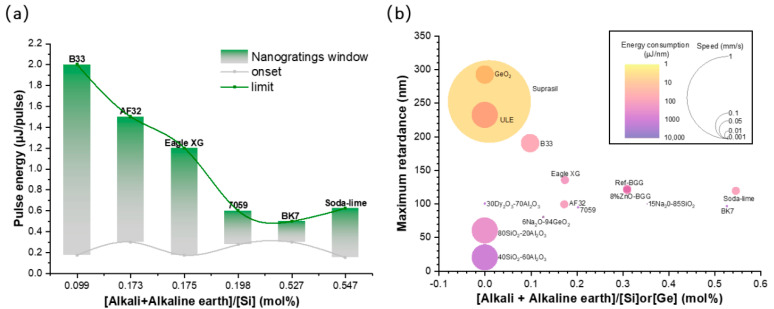
(**a**) NG processing windows at 50 kHz of alkali silicate glass samples and (**b**) bubble graph of the maximum retardance with increasing [alkali + alkaline earth]/[Si] or [Ge] cationic mol%. The Na_2_O-SiO_2_ and Na_2_O-GeO_2_ samples were extracted from [25] and [10], respectively.

The presence of alkali and alkaline earth cations can affect the NG formation [24,25,37,38]. From Figure 5a, it can be clearly seen that NG window at 50 kHz of the alkali silicate samples consistently decreased as the [alkali and alkaline earth]/[Si] cationic ratio progressively increased, with the exception of the soda-lime glass. One can note that the onset of the NG formation remained quite similar for all of the investigated glasses, whereas the upper boundary in terms of energy decreased when increasing the [alkali and alkaline earth]/[Si] cationic ratio.

Then, in order to tentatively summarize the relative performances of all of these glasses and understand who was the “best performer”, three parameters, as a function of the [alkali and alkaline earth]/[Si] cationic ratio, were reported within a single graph (see Figure 5b): (i) the writing speed, which represents the potential for writing a high amount of optical components; (ii) the maximum retardance (a key parameter for birefringent applications); and (iii) the energy consumption in μJ/nm (laser energy μJ used to write one unit of retardance expressed in nm). In this graph, Suprasil was found to reach the highest *R* value (252 nm) with the highest speed (1 mm/s) and the lowest energy consumption (0.6 μJ/nm). In the Suprasil, ULE, and GeO_2_ glasses, the formation of NGs was more likely to occur with higher *R* and speeds (ULE: 232 nm, 0.1 mm/s; GeO_2_: 292 nm, 0.05 mm/s) and lower energy consumption (ULE: 6.9 μJ/nm; GeO_2_: 1.79 μJ/nm). BK7 and 7059 possessed the lowest *R* values (BK7: 96.16 nm; 7059: 94.34 nm) and required low writing speeds (0.001 mm/s), along with a high energy consumption (BK7: 285.98 μJ/nm; 7059: 251.75 μJ/nm). Finally, B33, AF32, and Eagle XG exhibited relatively high *R*-values at moderate speeds and energy consumption (B33: 190 nm, 0.05 mm/s, 8.41 μJ/nm; AF32: 99 nm, 0.01 mm/s, 16.08 μJ/nm; Eagle XG: 135 nm, 0.01 mm/s, 25.93 μJ/nm). This trend is in agreement with the NG formation window. Other samples reported in the literature have been added in Figure 5b. The 15Na_2_O-85SiO_2_ [25], 30Dy_2_O_3_-70Al_2_O_3_ [39], and 6Na_2_O-94GeO_2_ [40] samples exhibited a quite useful *R*, but had the highest energy consumption and low writing speeds. Then, even if the speed was quite high for 80SiO_2_-20Al_2_O_3_ and 40SiO_2_-60Al_2_O_3_ [41], they exhibited low *R* with a quite high energy consumption while there were no alkali and alkaline earth cations. Although 80SiO_2_-20Al_2_O_3_, 40SiO_2_-60Al_2_O_3_, 30Dy_2_O_3_-70Al_2_O_3_ and 6Na_2_O-94GeO_2_ possessed lower alkali and alkaline earth cation contents, more energy consumption was required. This is again a clue that viscosity should be the key factor here. The BaO-Ga_2_O_3_-GeO_2_ (BGG) glass [42,43] exhibited a higher *R* and speed, but lower energy consumption. When the content of the alkali and alkaline earth cations increases, much more energy consumption is required for the formation of NGs.

## 4. Discussion on Nanopores Formation and Erasure

While this paper mostly focused on Type II, and an extensive discussion of all types of transformation was out of the scope of this work, some explanations on the mechanism that drives the NG formation are emphasized below.

At first, some fluctuations of the dielectric constant at the nm scale were proposed to trigger the process. In this transient nanoplasmonic model [7], this led to the formation of spherical nanoplasma hot spots induced by localized multiphoton ionization initiated by some seeds. The latter can either be defect centers, or glass free volume and voids initiated by the first pulse(s). In this model, these nanoplasma hot spots would experience an asymmetric growth oriented preferentially in the direction perpendicular to the laser polarization, provided that the sub-critical plasma density is reached.

Then, there is a sub-wavelength structuration of the plasma, creating an interference-like pattern or self-organized mechanism with a pseudo-regular array of high-density plasma nanolayers. The latest modeling works suggested that this “self-organization” originated from the above-mentioned heterogeneities of the dielectric constant initially present in the glass (or initiated by the first pulse), leading to multiple scattered wave interference, and hence the formation of a standing wave [44]. In this model, subsequently refined by Rudenko et al. [45], the grating formation process is reinforced by a pulse-to-pulse effect, resulting in a decrease in the NG pseudo-periodicity much lower than *λ*/2*n*, which scales with the increase in the concentration of the heterogeneities.

Finally, inside the plasma dense nanolayers, the formation of nanopores was observed in most oxide glasses, as widely described in this work. In our early work [10], the birth of these nanopores was associated with a tensile stress-assisted decomposition and a “soft” Coulomb force necessary to overcome the oxygen binding energy and to form nanopores upon an intense stress field. This was recently revisited as a tensile stress assisted nanocavitation process [11,12]. However, this is a plasma-mediated nanocavitation occurring within both thermal and stress “confinement conditions”, which can thus imprint nanopores at the “image” of the plasma spatial nanostructuration. This explains the formation of polarization dependent nanopores (i.e., spherical when using circular polarization but ellipsoidal when using linearly polarized light) [36].

**Figure 6:**
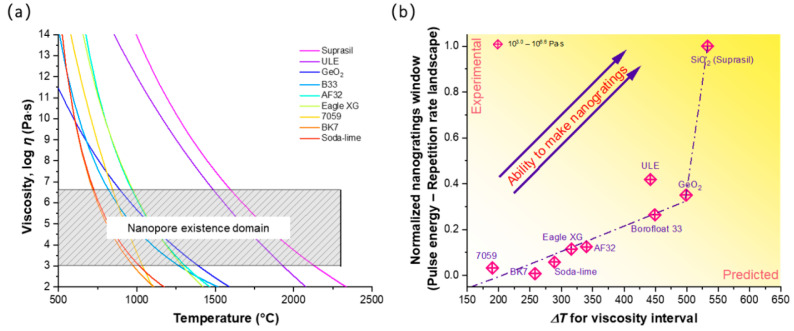
(**a**) Viscosity as a function of temperature for a variety of commercial and typical glasses, along with an estimated domain of NG existence from *T_soft_* (*η* = 10^6.6^ Pa⋅s) to *T_max_* (*η* = 10^3.0^ Pa⋅s). (**b**) Normalized NG window as a function of the temperature interval (Δ*T*) corresponding to the previously mentioned viscosity interval.

The above results provide additional clues that the formation of NGs is also highly impacted by glass viscosity, as suggested in a previous work [11,46]. According to the aforementioned cited references, the domain of existence of the nanopores (and thus NGs) was predicted using a simple approach following viscosity-based arguments [12]. The viscosity profile as a function of temperature for all of the oxide glasses investigated in this work, together with the estimated domain of NG existence, are reported in Figure 6a. The domain of NG existence was predicted by taking into account two boundaries in terms of viscosity, which can be then translated in terms of two limiting temperatures. The lower limit corresponds to the temperature, *T_soft_*, at which nanocavitation in the glass can occur and for which the viscosity value (*η*) is situated at around ~10^6.6^ Pa⋅s, forming nanopores that compose the NGs. The upper temperature limit, *T_max_*, relates to either the collapse or unstable hydrodynamic growth of the nanopores, resulting in the disappearance of the nanopores, hence the NGs. This corresponds to a viscosity value typically around ~10^3.0^ Pa⋅s, and was determined using the Peclet number (ratio between the viscous deformation and diffusion rates) as a local indicator for nanopore erasure [12].

The domain of the NG existence was thus predicted by taking into account the temperature difference between the *T_max_* and *T_soft_*, respectively, the upper and lower limits, and then compared to the experimental data in the energy—repetition rate landscape. In this view, the “normalized NGs experimental window” was used, that is defined as the integrated area of the Type II domain in Figure 3 divided by the one of SiO_2_ taken as the reference. Figure 6b shows a marked reduction in the normalized NG experimental window (normalized window area from Figure 2) as a function of the predicted temperature interval (Δ*T* = *T_max_* − *T_soft_*) between the two above-mentioned viscosity values. A strong link was found here and supports the previously reported work and the above-reported results.

## 5. Conclusions

In this work, the NG formation windows in a wide range of commercial oxide glasses were addressed in an energy—repetition rate laser parameter landscape and thoroughly characterized by polarizing optical microscopy and SEM analysis. The pure silica glass (Suprasil) and the alkali-rich borosilicate glasses (7059 and BK7) showed the largest and the smallest NG formation windows, respectively. The NG formation windows of the ULE, GeO_2_, B33, AF32, and Eagle XG lay between these two limits and progressively exhibited decreasing values. In agreement with the literature, the alkali and alkaline earth contents, closely connected to the glass viscosity, were proven to play a key role in the formation of NGs. The NG formation window decreased with the increase in the alkali and alkaline earth contents, which appeared to be correlated to the reduction in the predicted temperature difference determined from the “allowed viscosity interval”. The results agree with the literature and the proposed viscosity approach based on a nanocavitation process [12]. Although the soda-lime, BK7, and B33 glasses exhibited higher birefringence, the pure silica glass remained the “best performer” (high writing speed, low energy consumption, high retardance) compared to other oxide glasses. Future work includes the simulation of the temperature elevation during the irradiation process for each glass composition in order to predict the processing windows and exploitation of the Rayleigh–Plesset model to predict the lifetime of the NGs.

## Figures and Tables

**Table 1:** Name, type, and chemical composition of all of the glasses investigated.

Commercial Glass Name	Type	Chemical Composition (mol%)
SuprasilCG	Silica	100 SiO_2_
ULE	Titanium silicate	94.25 SiO_2_, 5.75 TiO_2_
B33	Free/low alkali aluminoborosilicate	81 SiO_2_, 2 Al_2_O_3_, 13 B_2_O_3_, 4 Na_2_O/K_2_O
AF32		66.43 SiO_2_, 11.28 Al_2_O_3_, 10.73 B_2_O_3_, 5.30 CaO, 4.63 MgO, 1.36 BaO, others < 1
Eagle XG		65.71 SiO_2_, 11.10 Al_2_O_3_, 11.65 B_2_O_3_, 8.64 CaO, 2.28 MgO, others < 1
7059		63 SiO_2_, 8.5 Al_2_O_3_, 16 B_2_O_3_, 12.5 BaO
BK7	Alkali borosilicate	69.13 SiO_2_, 10.75 B_2_O_3_, 3.07 BaO, 10.40 Na_2_O, 6.29 K_2_O, others < 1
Soda-lime	Soda-lime silicate	72.6 SiO_2_, 13 Na_2_O, 8.8 CaO, 4.3 MgO, 0.6 Al_2_O_3_, 0.3 K_2_O, 0.2 SO_3_, 0.1 Fe_2_O_3_
GeO_2_	Germania	100 GeO_2_

## Data Availability

Not applicable.
